# High immunisation coverage but sporadic outbreaks of vaccine-preventable diseases: the structural gaps in vaccination uptake in central highlands, Vietnam

**DOI:** 10.1186/s12889-025-23486-6

**Published:** 2025-07-03

**Authors:** Thanh Ha Nguyen, Thi Phuoc Loan Mai, C. Louise Thwaites, Jennifer Ilo Van Nuil, Mary Chambers

**Affiliations:** 1 Oxford University Clinical Research Unit, 764 Vo Van Kiet Street, Hochiminh City, Vietnam; 2 Centre for Disease Control Daklak, 72 Le Duan Street, Ban Me Thuot City, Vietnam; 3 Centre for Tropical Medicine and Global Health, Oxford, OX3 7LG UK

**Keywords:** Vaccine hesitancy, Structural vulnerability, Vaccine-preventable diseases, Social marginalisation

## Abstract

**Background:**

Despite the successful implementation of the Expanded Programme on Immunization (EPI) for the past three decades, Vietnam has recently witnessed outbreaks of vaccine-preventable diseases, indicating a potential gap in immunisation uptake across population groups. Daklak province is a rural highland area, home to 46 ethnic groups with complicated socio-economic backgrounds. The province reported sporadic outbreaks of vaccine-preventable diseases and low vaccine uptake in some remote low-socioeconomic groups despite a high record of provincial coverage. Within this context, we aim to explore the perspectives and experiences of ethnic minority communities related to EPI vaccination and how socio-economic and contextual factors influence such views and practices in Daklak province.

**Methods:**

We used qualitative data collected between 2018 and 2022 from in-depth interviews, focus group discussions, and participant observation with different stakeholders of the EPI programme. The study took place in nine districts across 25 communes of the province with different socio-economic characteristics and vaccination patterns. We invited mothers who were the primary caregivers taking children to vaccination and healthcare workers who were directly involved in vaccination delivery in the local areas. We incorporated the SAGE’s public health framework of the vaccine hesitancy matrix and the anthropological concept of structural vulnerability to discern the structural roots of vaccine attitudes and behaviours of the community.

**Results:**

Overall, the research shows that views and behaviours related to children’s vaccination are complicatedly influenced by multi-ecological factors. In particular, we found a critical influence of socioeconomic conditions and social networks on the community’s vaccine acceptance and uptake. The community’s interaction with the health system and the government through local healthcare workers was also critical in fostering community trust towards vaccines and the EPI programme. In addition, we revealed that the issues with non-compliance to the EPI in the lowest-uptake communities were structurally related to their economic vulnerabilities and social marginalisation.

**Conclusions:**

These findings implicate the need for tailoring public health and socioeconomic interventions to enhance vaccination opportunities in the marginalised groups.

**Supplementary Information:**

The online version contains supplementary material available at 10.1186/s12889-025-23486-6.

## Background

Vaccines are one of the most successful public health interventions, saving millions of lives every year and contributing to the reduction and elimination of many infectious diseases, such as polio and smallpox [1, 2]. However, in recent years, outbreaks of vaccine-preventable diseases have emerged in all parts of the world, with nine of the ten countries with the highest incidents of measles are low- and middle-income countries (LMIC), accounting for 74% of new infections [3]. An emerging concern from these outbreaks is the decline in immunisation uptake, even with established programmes such as routine childhood immunisation. The COVID-19 pandemic also caused significant disruptions to routine immunisation programmes, which contributed to a global decline in vaccination coverage for children, for example, a 33% reduction in Diphtheria-Pertussis-Tetanus 3rd dose globally in 2020 [4].

Under-vaccination and non-vaccination in LMICs are significant public health concerns despite the general high acceptance of vaccines in these countries [5]. Recent literature has identified that in LMICs, emerging antivaccine and hesitant sentiments are leading to delays or dropout of vaccination schedules, while there remain pragmatic constraints for communities to access vaccination in many areas with poverty [2, 5, 6]. Programmes with proven significant success, like the Expanded Programme on Immunization (EPI), still encounter challenges in sustaining high immunisation coverage for children, especially for those in marginalised and poor areas, as well as challenges in ensuring stable delivery systems [7]. However, data on these challenges remain limited [8].

In Vietnam, the national immunisation coverage for childhood vaccines has remained high since 2010, with a high reported index of vaccine confidence [3, 9]. However, in the past few years, Vietnam has suffered outbreaks of vaccine preventable diseases, including recent measles outbreaks across the country in 2024 [10, 11]. This implicates a potential gap in childhood vaccination throughout the country. Adverse events related to immunisations were documented to be among the main concerns related to parents’ vaccine hesitancy, originating from several cases of sudden deaths following Hepatitis B vaccination and 5-in-1 vaccination in young children between 2012 and 2013 [12, 13]. Some studies showed that exposure to news covering these incidents reduced parents’ intentions to have their children vaccinated [13, 14]. On the other hand, low vaccination records were reported in children of households with low socioeconomic status or belonging to ethnic minority groups [15, 16]. However, most of the current evidence is quantitative and cross-sectional, making it impossible to discern how socioeconomic factors affect low vaccine uptake in these communities. Within this context, this paper presents qualitative data collected between 2018 and 2022 in Daklak Province, a rural area in the Central Highlands of Vietnam, that explores mothers’ views and practices on childhood vaccination. Overall, the province maintained high coverage of childhood vaccination rates over the years but since 2017 they have witnessed sporadic outbreaks of vaccine-preventable diseases in marginalised ethnic minority communities, suggesting a gap in immunisation uptake throughout the province [17, 18].

Methods.

## Research setting– Daklak province, Vietnam

Daklak province is a mountainous area, with natural forests extending over 45% of the province area. With the majority of the population (73.64%) living in rural areas [19] the province experiences a disparity in socioeconomic conditions among the population, with ethnic minorities accounting for the highest proportion of poor households [20]. Daklak province is inhabited by 47 ethnic groups residing in both urban and remote mountainous areas. They practice different traditions, speak different languages, and have different religious beliefs. The 47 groups are often classified into three major categories: 67% of the provincial population are Kinh people– the dominant Vietnamese ethnicity, 20% are indigenous groups to the province, and 13% are migrant ethnic groups [20]. With such a diverse ethnic population, Daklak province is often considered one of the most socio-politically complicated areas in Vietnam. During the French colonial administration (1884–1945) and the US-Southern Vietnam administration (1955–1975), the province was a key area for economic development and military activities due to its rich natural resources and location at the centre of the country and the Indochina peninsula [21]. Plantations were constructed and many indigenous groups lost their native lands and became labourers on these plantations [21]. After Independence (1975), there was a mass migration movement to the province by both Kinh people and Northern ethnic minority groups as people looked for economic opportunities after this prolonged period of war [20]. This had led to a significant reconfiguration of the indigenous social structure, sometimes leading to tension [22].

The EPI was widely implemented in Daklak province and the Central Highlands region between 1989 and 1990 [23]. Overall, the provincial EPI coverage remains high thanks to extensive efforts of outreach vaccination activities of local commune health centres to remote communities in the mountains. However, data provided by Centre for Disease Control Daklak revealed that the uptake rates were still low in some areas, particularly those with large ethnic minority populations. Quantitative studies showed that mothers belonging to ethnic minority groups and low education levels had lower knowledge of vaccines and lower rates of maternal and childhood vaccination [24, 25]. During 2007–2013, the province witnessed sporadic cases of vaccine preventable diseases such as neonatal tetanus and Japanese encephalitis while being affected by a diphtheria outbreak in 2020 and a measles outbreak in 2024 [17, 18].

### Theoretical frameworks

To understand views and practices related to vaccines and vaccination, a wide range of theoretical frameworks and models have been developed [26, 27]. In this paper, we adopted the SAGE matrix of vaccine hesitancy (Table 1) to explore different domains influencing caregivers’ opinions and experiences related to childhood vaccines and vaccinations. The SAGE Working Group’s Matrix of Vaccine Hesitancy Determinants categorises influence factors in three areas: [1] individual/group factors regarding perceptions and experiences about vaccine and disease prevention [2], contextual influences, e.g., historical and socioeconomic context or geographical living conditions, and [3] vaccine- and vaccination-specific factors, e.g., the design of vaccination programmes [28].

**Table 1:** The SAGE working group determinants of vaccine hesitancy matrix (WHO, 2014)

**Individual factors/Group influence**	Attitudes, and knowledge about vaccines Previous experiences with vaccination Attitudes and beliefs about health and disease prevention Perceived risks about diseases risk and severity Experiences with healthcare workers Trust in the system delivering vaccination programmes Social influence on vaccination
**Vaccine-specific issues**	Implementation of the vaccination programme Introduction of a new vaccine Mode of administration (injection/oral) Vaccine supply Cost related to vaccination Vaccination schedule
**Contextual issues**	Information and media about vaccines Opinions about vaccines of influential leaders/key-opinion leaders Religion/culture/socioeconomic conditions Policies about vaccination Geographic barriers

Recognising criticism of this model’s ability to discern the mechanisms of how these factors influence the opinions and practices related to vaccines [26], we added an anthropological lens of structural vulnerabilities to explore how historical, political, and socioeconomic contexts shape the life conditions that facilitate such patterns of vaccine attitudes and behaviours. In this concept, the vulnerability of an individual or group to adverse health outcomes is influenced by more extensive structural processes, such as policies and legislation related to race and ethnicity [29] and/or the disparity in the allocation of resources [30]. It has been used in studies investigating unequal burdens of illness and injuries in immigrants in the US [29, 31], morbidity in communities without means of production [32], and susceptibility to COVID-19 infections for poor and homeless people [33]. In this paper, we applied the concept of structural vulnerability by referring to economic vulnerabilities that reduced the opportunities to take children to vaccination, and social marginalisation that prevented tailoring support for these under-vaccinated communities.

### Data collection

Data in this study were extracted from two mixed-method studies conducted between 2018 and 2022. The first examined barriers to childhood vaccination in ethnic minority communities and was followed by supplementary vaccination activities in the villages with the lowest uptake of vaccination in the province between 2018 and 2019. The second study explored the vaccination experiences of communities and healthcare workers within the context of the COVID-19 pandemic between 2020 and 2022. The research design for the second study was based on a preliminary analysis of the first study. This enabled us to explore the vaccination situation in the local context more in-depth and particularly expand to discover patterns that emerged from the first study. Question guides for the second study were constructed based on the preliminary findings of the first study, the SAGE’s framework, and the concept of structural vulnerability to understand how the socio-economic and political contexts affect the community’s attitudes and uptake of childhood vaccination at different ecological levels. This paper reports qualitative data from semi-structured in-depth interviews (IDIs), focus group discussions (FGDs), and participant observation collected in 25 communes across nine districts in the province. Topic guides for the interviews and focus group discussions with the community for both studies are provided as Supplementary files [1–3]. IDIs took place in different settings (e.g. at the community health centres or at the participants’ houses) depending on participants’ preferences and convenience. During the pandemic, due to social restrictions, interviews were conducted by telephone. All FGDs took place before the COVID-19 pandemic in the community health centres which are often located in the centre of a commune. IDIs and FGDs were conducted in Vietnamese, but in the FGDs, there were local translators to support the discussion. For participant observation, the first author (HN) travelled to the province over a four-year period, often s attending the community health centres. HN observed multiple vaccination days, both at the health centres and inside remote villages, and paid multiple visits to the communities, including visiting specific households with children having no or very few records of vaccination.

### Participant recruitment

We invited caregivers whose children were eligible for EPI vaccination and healthcare workers at village and commune levels who were involved in vaccination delivery to participate in the study. For IDIs and FGDs, we only invited mothers as they were the main caregivers taking children to vaccination in the local area. We held informal conversations with grandmothers taking children to the commune health centres on vaccination days. We used purposive sampling to include mothers of different ethnicities and socio-economic conditions, and mothers of children with different patterns of vaccination (non-vaccination, incomplete EPI schedules, and completed EPI schedules). We also invited healthcare workers who were involved in delivering the local EPI to the communities, particularly village health workers and the vaccine coordinators of each community health centre, to gain different perspectives on the phenomenon. Participant recruitment was supported by Daklak CDC and local healthcare workers, who helped us identify and invite potential participants to the research.

### Data analysis

We followed the framework analysis approach [34] as guidance to analyse data for this paper. All FGD and IDI recordings were transcribed in Vietnamese by contracted collaborators and reviewed by the research team. To protect participant’s identities, participants’ names and locations, including villages, communes and districts, in the manuscript were pseudonymised. Transcripts, field notes, and documents were entered into Nvivo 12 SQR for analysis with password protection. During data collection, the first author (HN) listened to recordings and made summaries while noting down themes and information, especially any new findings, to explore in later interviews. Data familiarisation happened simultaneously with data collection. An analytical framework was built based on the SAGE’s matrix of vaccine hesitancy determinants. HN coded the whole data set while having biweekly discussions with other authors about coding and data interpretation. To construct themes from the data, we drew a conceptually clustered matrix [35] to discern different categories of influencing factors, from which we would explore the relationships among categories and across participants. Data triangulation was conducted with different sources (different groups of participants) and different collection methods [36] which allowed us to cross-check data while increasing the comprehensiveness of findings [37]. When analysing data about vaccine hesitancy, we identified a disparity in information reported by different participants concerning the reasons why certain groups have lower vaccination uptake than others. We report all perspectives in the Result sections along with an interpretation into such differences.

## Results

Table 2 summarises the participants’ demographic information of those who participated in IDIs and FGDs. We recruited 71 mothers, including 18 Kinh people, 45 belonging to indigenous groups, and eight belonging to migrant groups, and 54 healthcare workers, including 28 village health workers, 20 vaccine coordinators of commune health centres, and six other health centre staff.

**Table 2:** Summary of participant demographics in in-depth interviews and focus group discussions

		Community members	Healthcare workers
**Socioeconomic characteristics**	Kinh groups	18	30
	Indigenous groups	45	22
	Migrant groups	8	4
**Urbanicity**	Rural commune	53	10
	Urban commune	18	44
	Live/Work in marginalised villages	32	28
**Occupations**			
	Village health workers	N/A	28
	Commune vaccine coordinators	N/A	20
	Other health centre staff	N/A	6
	Farmer	55	N/A
	Labourer	4	N/A
	Home business	4	N/A
	Stay home	8	N/A
**Total**		**71**	**54**

**Fig. 1:**
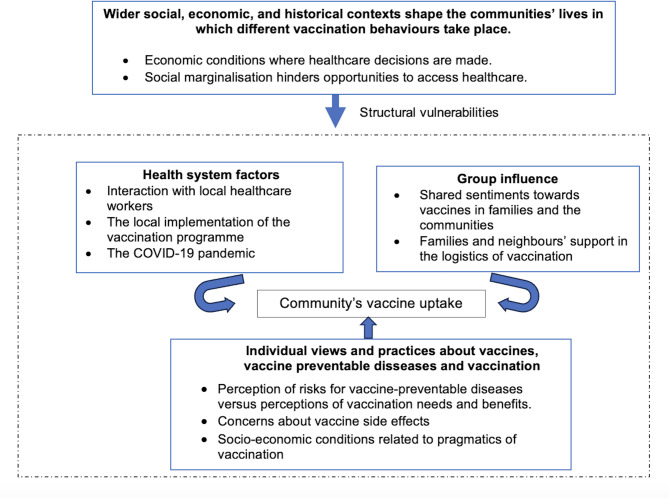
Factors influencing vaccine views and behaviours of ethnic mothers in Central Highlands, Vietnam

Immunisation records collected at community health clinics and Daklak CDC showed that childhood vaccine immunisation coverage was generally high at the province and commune levels. However, through fieldwork, we observed a difference in vaccine uptake rates among ethnic groups with different socioeconomic conditions. For example, indigenous groups like the Ede living in urban and suburban areas, and several Northern migrant groups had high rates of childhood vaccination. Meanwhile, more peripheral groups such as indigenous Mnong, remote Ede groups or migrant Xe Dang and H’Mong groups had very scant uptake. Often, these groups lived within villages located within communes with relatively high uptake rates, masking their under-vaccination status to public health authorities. However, vaccine preventable disease outbreaks were often identified in unvaccinated individuals in these villages. To explore why people did or did not take their children to vaccination, we adapted the SAGE framework to describe different factors that may have encouraged or hindered mothers’ views and practices towards childhood vaccination at individual, social, and health system levels. In addition, we portrayed the structural processes that accounted for the immunisation disparity in marginalised villages (Fig. 1).

### Individual views and practices about vaccination

#### Perceptions of risks for vaccine-preventable diseases versus perceptions of vaccination needs and benefits

Regardless of their attitudes towards vaccines, mothers acknowledged the risk of diseases for their children. However, they mostly had vague ideas about which diseases vaccines protect against and the mechanism of action. For those who could name diseases, the most frequently reported diseases were tetanus, measles, diphtheria, and Japanese encephalitis; these were also the diseases which caused recent outbreaks in the province. This suggested that their awareness was heightened when outbreaks occurred and there were extra attempts by local CDC to increase immunisation coverage with improved communication and booster vaccination campaigns.

I just saw measles on the television. People who were unvaccinated then got measles. So many cases on TV, from mothers to children, and they found out those [infected] were not vaccinated. This morning, upon check-up, [health centre staff] told me that my son had not received any measles vaccine yet…oh measles… so scary. And then, she [village health worker] called me to vaccination. I was so glad. I came immediately for [my son] measles vaccination.

(Lam, a migrant mother)

There was a difference in the perceived need for vaccination to prevent diseases. Mothers with children being fully or partially vaccinated acknowledged vaccine benefits. Some expressed their active demands for their children’s vaccination by asking local healthcare workers or looking up vaccine information on the Internet. Some had their children immunised privately, with vaccines brands not provided by the EPI programme. However, mothers with non-vaccinated children often disregarded the necessity for vaccination, reporting a preference for a natural lifestyle in which they accepted the life god would give to them or rationalising that the older generations had no vaccinations but still survived for a long time. However, we observed that these same mothers were also not socialising with other people in the village or the commune and were not taking part in any social activities, implying that their rejection of children’s vaccination may be embedded in a broader disintegration from the wider communities.

#### Concerns about vaccine safety

Concerns about vaccine side effects were reported by mothers regardless of their vaccination patterns. When asked, they could tell me that the most common side effects of vaccination were fever, pain, and swelling and explained how to handle these side effects. They reported being repeatedly instructed by local healthcare workers about vaccine side effects, while some also actively searched on the internet for more information.

Side effects were also frequently reported as reasons for mothers delaying their children’s vaccination or dropping out from EPI schedules. This was due to their children experiencing severe reactions that required medical intervention or exposure to news about adverse events.

Many other kids in the village had the same symptoms. They received different vaccines, but they all had very high fevers. I did not dare continue my children’s vaccination. I would not have taken children to vaccination in the first place if I had known before [about fever]. The healthcare worker said it would be okay, but the children got such high fever.

(Lam, a migrant mother)

Many mothers recalled an event in 2012 and 2013 when there were fatalities associated with 5-in-1 vaccines administered in other provinces that sparked extensive media coverage. Some also showed hesitancy when they heard rumours about adverse events from relatives living in neighbouring villages and communes.

Every year, my commune has good vaccination coverage. But that incident affected uptake…the child died in the Ea Ping commune…they are relatives of people in my commune. So they shared the news with each other.

(Phuong, an ethnic vaccine coordinator)

It is critical to note that the communities often associated the EPI programme with social policies from the government. Many ethnic mothers perceived the EPI as a social benefit the government offers to improve the community’s lives. Thus, their attitudes towards EPI vaccines may have been influenced by the sentiments of the communities towards the government. Trust in the government’s intentions helped reinforce trust in vaccines and the vaccination process against the increasing uncertainties about vaccine safety during the adverse events. For example, one mother commented about vaccine side effects:

I think it’s…they [the government] must have assessed vaccines before delivering to the communities. There is no way they give us the fake one.

(H’Pong, an Indigenous mother)

#### The pragmatics of vaccination convenience

Socioeconomic conditions were frequently reported by participants as a factor hindering vaccination. Children in the poorest households were often not vaccinated or severely under-vaccinated. Mothers explained that their family only had one motorcycle, often used by husbands to go to work. In many families, both parents and even grandparents had to go to work as they lived on a daily income. It became challenging for them to take absences from work to take children to vaccine appointments and look after them with vaccine side effects.

HN: “why did you stop bringing your child to vaccination?”

*H’Kim: “I don’t have time. I am busy with my labour work since 3a.m. I only return in the evening.”*

*HN: “So you are busy working and cannot take your child to vaccination. If you are absent from work for one day*,* will it affect you a lot?”*

*H’Kim: “One day off means I lose one day of income. It’s not that I make a lot*,* but losing one day of income still means losing money so…”*.

(H’Kim, an Indigenous mother)

On the other hand, there were marginalised villages located too far from community health centres for the community to take their children to vaccination. Many remote villages still had unpaved roads making it hard for mothers to carry small children on their motorbikes, especially in rainy seasons. While there were places where community health centres implemented mobile vaccination in remote villages to ease the convenience of travelling, this was not a common practice throughout the province, which will be explained below.

### The influence of social networks on vaccine acceptance and uptake

Family and neighbours played an essential part in attitudes and behaviours related to vaccines. In villages with high vaccination rates, childhood vaccination has become a social tradition, in which new mothers naturally accepted vaccines as “everyone is doing it.”

*“In my village? For children*,* I think it’s like a tradition from generation to generation. If a child is born*,* he/she has got to receive vaccines. They probably think like me*,* vaccination is to prevent diseases. “*.

(H’Pong, an Indigenous mother)

We also observed a strong influence of family members and peers in the case of hesitancy. For example, when mothers were concerned about side effects, they often looked to their peers to decide whether to continue vaccinating their children. In other cases, we observed that in households where children dropped out of the EPI schedules, mothers reported being influenced by the attitudes of husbands or extended families. For example, when being asked for the reason why she stopped bringing her child for vaccination, one mother said:

Yeah, my husband saw my child having a high fever, so he asked to stop bringing the child to vaccination temporarily.

(H’Ana, an Indigenous mother)

On the other hand, strong social networks can facilitate the logistics of taking children to vaccination. In villages with high vaccination uptake, neighbouring mothers reminded each other of vaccine schedules. When someone did not have a vehicle to bring children to vaccination, others provided her and her baby a ride to the health centre. In contrast, in villages with very low uptake rates, the bonding between mothers was fragmented, possibly because they had to prioritise working and lacked the time for socialisation. As a result, childhood vaccination was hardly discussed among mothers.

No, I don’t know. I rarely asked [my neighbours] about vaccination. Well, I hardly talk to neighbours. We all have to go to work.

(H’Ban, an Indigenous mother)

### Influence of health system factors related to vaccination

#### Interaction with local healthcare workers

Similar to existing evidence, in this research setting interactions with local healthcare workers had a strong impact on mothers’ acceptance and compliance to vaccination. Trust and familiarity with local healthcare workers were regarded as the key factors for mothers to accept vaccines and lessen their worries over side effects. Village health workers, who were also community members, were often regarded as pivotal in persuading and encouraging mothers to accept vaccines and comply with vaccination schedules. Some mothers reported that they relied entirely on village healthcare workers to remind them to take their children to vaccination:

With the first child, I didn’t know about vaccination or anything else, I didn’t hear anything about that. No one came to my house about vaccination or anything. I didn’t hear, I didn’t know. Only for the second child I received an invitation letter sent to my home.

(H’Ngoan, an Indigenous mother)

For healthcare workers at the community health centre, mothers reported that friendliness and enthusiasm were important to keep mothers coming for children’s vaccinations. Trust in HCWs was also critical in reassuring and explaining to parents about AEFIs.

HN: “were you aware of those side effects before your child’s vaccination?”

H’Yen: “I knew it. But it was my first child. I was very nervous.”

*HN: “How about now?”*

*H’Yen: “Now I feel much relieved. If my child has a fever after vaccination*,* now I know I can just go to commune health centre for his medication. They [commune health centre staff] explained to me*,* so I feel less worried.”*

(H’Yen, an Indigenous mother)

It is important to note that trust in local healthcare workers was intertwined with trust in the government they represented government officials. Thus, ethnic marginalised villages with very low uptake rates tended to have tension with the government and resided remotely from the commune centre. We also observed a strong reservation when ethnic mothers communicated with local (Kinh) healthcare workers.

#### How vaccination is locally delivered

In this context, we found that the local implementation of the EPI in each location could impact the uptake of childhood vaccination. Due to the concerns over adverse events, healthcare workers were very cautious in screening children to decide whether the child was fit enough to receive vaccines. They may delay vaccination if children had light illnesses such as sore throats or coughing. Many mothers also shared with us that because of this, mothers might decide not to bring children to vaccination if they felt that the child was sick or felt warm, especially for those who thought that they had ‘wasted’ time off work with failed trips to the health centres. Repeated missed opportunities were often cited as a reason why children missed their timely vaccine appointments.

Another factor that may have affected the convenience of children’s vaccination for mothers in remote villages was the implementation of mobile vaccination delivery. There were no compulsory national guidelines about mobile vaccination, and the local health centres made their own decisions on whether to conduct mobile vaccination in remote villages. We found that this practice was not universal across the province but depended on human resources and infrastructure that enabled mobile vaccination. In some communes where community health centres were located too far from households along unpaved roads and there was no mobile vaccination, many mothers lacking transportation reported not being able to take their babies for vaccination. This is illustrated in this quote of an ethnic mother whom I met during a booster vaccination day held in the village community house. She shared that usually, she could not take children to the routine vaccination at the commune health centre due to distance:

“*If they vaccinate here [within the community house]*,* then I will go. If it’s at the CHC [3km from her house]*,* I won’t be able to go. Too far. Probably the same with some older mothers.*

(H’Muon, an Indigenous mother)

#### The impact of the COVID-19 pandemic

During the COVID-19 pandemic, the Vietnamese government implemented rigorous responses to contain the outbreaks. There were periods of local and national lockdowns when there were outbreaks. These lockdowns prevented mothers from taking children to vaccination. As the health centre staff were the main workforce to tackle the pandemic at the commune level, mothers perceived health centres as high-risk transmission places, which discouraged them from going there.

On the other hand, the prioritisation of delivering COVID-19 vaccination meant that the local healthcare workers spent less effort on reminding and encouraging mothers to access childhood vaccination, while mobile vaccination was suspended in many villages. As a result, there was a drop in immunisation coverage in 2020 and 2021.

For perceptions and attitudes towards EPI vaccines after the emergence of the COVID-19 pandemic, most interviewed participants, including both healthcare workers and mothers, reported no vast difference in the attitudes towards EPI vaccines, citing that in the province, most community members were already supportive of children’s vaccination prior to the COVID-19 pandemic. However, some mothers told me that they actively called village health workers to ask for children’s vaccine appointments instead of waiting to be reminded as before. One village health worker noticed with me that more people were bringing their children to vaccination after the pandemic:

*H’Chan: “After the pandemic*,* I’ve seen they took children more often to vaccination*,* maybe 100% [of the children scheduled for vaccination] went.”*

HN: “what made them change, in your opinion?”

*H’Chan: “Because before*,* their children hadn’t got any vaccines*,* but there was no outbreak of diseases. So they may be less concerned for their children. They said that even without vaccination*,* their children were fine*,* nothing happened. But when the [COVID-19] outbreak happened*,* they’ve seen that without vaccination*,* they wouldn’t have immunity. So the children may be more likely to be sick*,* maybe die.”*

(H’Chan, an Indigenous village health worker)

These findings suggested that the community in this province may perceive a greater need for general vaccination when witnessing the outbreaks of COVID-19.

### The structural roots of vaccination disparity in the Province

The under-vaccinated villages were often marginalised communities with economic deprivation and social separation from mainstream communities and local government staff, including health centre staff. These communities were either indigenous groups to Daklak province or undocumented migrant groups who moved to the province after the wars.

#### Economic vulnerabilities leading to inconvenience for childhood vaccination

As discussed earlier, households with financial disadvantages found it difficult to take children to vaccination. On exploring the roots of their poverty, we realised that while land was often considered an essential asset for the household economy, both marginalised indigenous and migrant groups did not possess any land. As a result, they had to move deep inside the forest to find new places to farm or seek cheap labour jobs. In both cases, this posed pragmatic difficulties for mothers taking their children to vaccination as they may have lived too far from the health centre, or they could not ask for days off to take their children for vaccination. In other cases, many young parents moved to big cities to work in industrial zones. Some brought their children with them, but there was no record of their children being vaccinated in their temporary residence. The ones who left their infants at home depended on their children’s grandmothers to keep up the vaccination schedule. These older women often reported logistical challenges in taking their small grandchildren to vaccination spots, such as no transportation or being unable to ride a motorbike. In the most marginalised villages, poverty was often extended within large families and, in many cases throughout the whole villages, making it impossible for close relatives and neighbours to assist in taking children to vaccination.

#### Social marginalisation in relation to acceptance of governmental vaccination programmes

Social marginalisation refers to the exclusion of certain groups from accessing resources and opportunities due to a specific characteristic such as ethnicity, gender, or socioeconomic status. In this research setting, social marginalisation was manifest in a sense of “othering” that differentiates the marginalised ethnic communities from the mainstream groups in the same commune. This inadvertently hampered the uptake of the EPI programme for marginalised communities who often lived separately from the mainstream groups.

When asked for opinions about the low-uptake villages inside a commune, members of mainstream villages and healthcare workers often attributed the low adherence to EPI vaccination and other communal activities to deliberate intentions. “*Too preoccupied with earning money”*,* “being indifferent to children’s health”*,* “no appreciation of vaccine benefits”*,* and “not wanting to integrate into mainstream lives*” were common phrases from interview data with the mainstream groups when describing behaviours of peripheral communities. These narratives reflected a tendency to criticise the marginalised groups for remaining “inward” and not learning from “more advanced” groups, from farming techniques and saving habits to appreciating health behaviours, including childhood vaccination.

However, data from these marginalised villages showed a more complex narrative. They were not entirely resistant to governmental programmes but showed more reservation if these activities were conducted outside their villages. This was illustrated in the interview with Ly Nin, a migrant mother, who expressed hesitancy to interact with healthcare workers at the community health centre but responded well to mobile vaccination inside her village conducted by the same staff:

*Ly Nin: “I don’t… I don’t feel used to going here [the community health centre]…I go to the pharmacy. It’s more comfortable for me to have conversations there.”*

*HN: “But I thought you still interact with them [health centre staff] during vaccination for your child.”*

*Ly Nin: “Yeah but only a few people. Like I interacted with these staff [pointing with her head to the vaccine coordinator sitting outside] in the village. But here*,* no. I rarely go.”*

Interviews with village health workers from other marginalised villages reported a similar pattern. Even when these villages had easy access to health centres, mothers were less likely to bring their children for vaccination. However, when the health centre started delivering vaccination inside the villages, more mothers participated more readily. This suggests that their lack of enthusiasm for complying with the EPI (or any other government activities) might not be related to vaccines or vaccination programmes. Instead, it may reflect hesitancy for the marginalised ethnic communities to engage in activities in a setting that lacks familiarity and cultural sensitivity. In this way, the sense of “othering” was internalised in marginalised communities, hindering their participation in activities that they perceived as “external” to their group.

## Discussion

This research resonates with findings from other studies that vaccine acceptance and uptake are influenced by various social, political, and systemic factors other than individuals’ knowledge and intentions. In addition, we showed that these factors are interrelated in hindering or promoting vaccine acceptance and uptake. For example, we pointed out that the influence of mothers’ social networks can either promote or hinder mothers’ confidence in vaccine safety. One mother may hesitate to continue children’s vaccinations upon hearing about side effects if surrounding mothers decided to do so. This is also observed in studies in both high-income and LMIC settings [26]. On the other hand, neighbours or other family members can help facilitate the convenience of taking children to health centres by giving rides to mothers without transportation, which was also reported in other resource-constrained settings [38]. The healthcare workers’ implementation of the EPI may also affect vaccination uptake. Delaying vaccination for fear of adverse events led to many missed opportunities. Due to health system regulations, if the children became older than the eligible age, they could no longer receive the EPI vaccines. In many communes, mobile vaccination inside remote villages reduces the travelling challenges for mothers. However, the decisions to deliver vaccination this way depends on individual health centres, which may explain for the difference in vaccination coverage among villages with similar demographic characteristics across the province. These findings implicate that the uptake of children’s vaccination is influenced by multiple social and systemic factors, Hence, it is crucial to consider the broader contexts in which parents perceive and make decisions about vaccines and vaccination.

We identified that for children with no or very few recorded vaccinations household financial hardship strongly influenced the logistical convenience of taking children to vaccination. In Daklak province, economic vulnerabilities of peripheral communities were reflected in different layers: the need to prioritise daily income for survival, precarious jobs such as farm labour, and movement from home to deep forests due to lack of land possession or migration to big cities for manual labour in industrial zones. Similar relationships between economic difficulties and the patterns of under-vaccination and non-vaccination were found in rural areas of other LMICs [8, 39–41]. In these settings, parents often had to set children’s vaccinations behind other economic activities to survive. Even when vaccines are offered free of charge, the indirect cost of transportation and loss of income during these vaccine appointments could hinder them from taking children to vaccination clinics. The 2023 UNICEF report about immunisation inequality concluded that poverty is at the centre of inequities in children’s possibilities to receive vaccination [36]. Data from the report shows that in LMICs, children belonging to the poorest 10% of households are much less likely to be vaccinated compared to those in the wealthiest 10% of households [36]. Thus, this research echoes the persistent recommendation that addressing poverty is essential to improving health behaviours and outcomes for marginalised communities.

Social marginalisation is another crucial factor reducing vaccination possibilities for peripheral communities. Many existing studies reported that in rural areas, “remoteness”, i.e., communities living far away from vaccination clinics, is one common pragmatic barrier to vaccination [42–44]. While we observed the same pattern of remoteness in peripheral communities, this research pointed out that the underlying reason for such remote residency is strongly related to social marginalisation. These under-vaccinated communities live deep inside mountainous areas and interact with reservation with governmental officials. Such communities often do not respond to governmental programmes, including the EPI. The sense of separation may be explained by the reconfiguration of the social structure in the province since the wartime. The colonisation and wars disrupted the societal system and livelihoods of the indigenous communities in the province [20, 21]. After Independence, the mass migration movements of other Northern groups added in the complicatedness to the social dynamics. These massive changes reportedly led to conflicts between different ethnic groups [22]. Thus, enhancing social cohesion among different groups may help enhance the marginalised communities’ participation in mainstream activities, including vaccination programmes.

Several studies similarly describe how political policies, and marginalisation may lead to disparity in vaccine uptake among different population groups [45–47]. For example, Hussain and colleagues [46] showed that the Hinduism in India has put the Muslim community into “minority” status, pushing them into urban slums with increasing distrust in the government. This paved the way for the decrease in polio vaccination uptake and subsequent rise in polio infections. Some studies pointed out that marginalised communities may reject vaccination programmes because they misalign with the communities’ needs, and the government should address other priorities instead [45–47]. Findings in this research add to the current evidence that to improve vaccine uptake to the communities where services are hardest to reach, we must also consider enhancing the trust between them and the government, as well as how the delivery of the vaccination programme can fit in within their socioeconomic conditions.

### Implications of this research

This study has several important implications for research and interventions targeting improving community vaccination coverage. First, health inequalities must be examined at local levels. While most literature often homogenises ethnic minorities to one group, these research findings reveal a discrepancy in the uptake among different ethnic minorities. Yet, only after incidents of vaccine preventable diseases did the gaps in their vaccination uptake reach the attention of public health authorities and the local government. Due to the administrative system of Vietnam, these peripheral villages may be in the same commune as other villages with higher socioeconomic status and high vaccination uptake and therefore managed as a whole. As a result, under-vaccination and non-vaccination in these villages can be under-detected by policymakers and public health authorities unless an incident of vaccine-preventable disease is identified. Such underrepresentation in the administrative system and in the literature can also contribute to inadequate attention and support for their life quality and health behaviours [48], including tailoring strategies to encourage children’s vaccination. This issue becomes more pertinent, given the missed opportunities of children’s vaccination during the COVID-19 pandemic between 2020 and 2022 and the shortage in the national supply of EPI vaccines in 2022 [49, 50]. These contextual factors were attributed to a decline in national vaccination coverage, which may have contributed to measles outbreaks in 2024 [51]. A recent survey in Ho Chi Minh City found that 30% of children in low-immunity areas were from immigrant families [52]. These families often lack residential registration with the local authority and thus may be excluded from public services, including EPI vaccination [53]. These patterns are similar to those observed in this study, which suggests a pressing need to identify and improve immunisation coverage for marginalised children in Vietnam.

While existing studies and dominant views about low vaccination uptake in marginalised communities often blame these groups’ unresponsiveness towards the governmental programme, this research suggested that marginalised communities would engage in government-proposed activities if these activities fit with their concerns. Prolonged periods of war triggered drastic economic disruptions to the province, depriving the poorest groups of land possession. As land is the main asset in the province, some communities lost their livelihoods and resorted to unstable jobs. Thus, it is necessary for the government to implement specific policies to improve the socioeconomic status conditions for these communities to enable life conditions where decisions related to vaccination are better facilitated. This becomes more critical within the context of COVID-19 pandemic as new evidence has shown that people with low socioeconomic status conditions are more disproportionately affected by the pandemic [54, 55]. In Daklak, poverty is likely to perpetuate among ethnic communities in the province since people in the lowest income group were reported to lose their jobs during the pandemic, making them more susceptible to precarious living conditions and challenging opportunities for children’s vaccination. Our research recommends that support policies for underserved communities include more criteria than merely the level of poverty. For example, the Ayushman Bharat programme in India, which aims to improve universal accessibility to health services, has extended its beneficiaries based on socio-economic deprivation criteria, rather than just the poverty line, potentially doubling the number of people covered by public health services [56].

This research echoes existing studies about the importance of social bonding among peer groups on mothers’ acceptance and uptake towards children’s vaccination. However, there seem to be limited interventions focusing on promoting a vaccination culture within the communities. Some intervention studies took into consideration the influence of social network but focused on having influential gatekeepers promoting vaccination to community members rather than targeting the social basis of vaccination in a community [26]. Thus, we strongly advocate for intervention in creating mutually positive sentiments about vaccines shared within parenting communities. On the other hand, this research shows that engagement with marginalised communities about vaccination can benefit from improving communities’ trust in the local authority and general government. This can be conducted through implementing specific socioeconomic policies for marginalised communities tailored towards their needs and cooperating with influential key members such as religious leaders or village leaders in the communities. There has been evidence that engaging the community in the development and implementation of public health programmes can be effective in improving programme quality [57, 58]. For example, India has implemented various initiatives that allow for civil participation in planning and monitoring health services at national, state, and district levels. These initiatives have been reported to increase the community’s awareness of service accessibility and improve maternal and neonatal health outcomes, including immunisation coverage, especially for marginalised groups [59]. A systematic review by Jain and colleagues (2022) found that in several studies, employing new community-based bodies for supporting local vaccination programmes, such as identifying and monitoring vaccination beneficiaries, showed consistent effects in improving children’s immunisation in LMICs [58].

### Limitations of the research

This study has several limitations. Firstly, there were gaps in the sampling strategy as we could not invite important groups from both the demand and supply sides, which may have impacted the uptake and delivery of the EPI. From the community side, we did not include family members who may have impacted the decisions in children’s vaccination, such as husbands and grandparents. We may have missed exploring their perspectives and experiences related to their grandchildren’s vaccination and, thus, unable to propose recommendations to address challenges for this group. Political sensitivities with marginalised groups meant that we could only spend relatively limited time inside the marginalised ethnic communities. Thus, findings related to ethnic communities’ lives were limited and from an etic point of view. This resulted in a limited understanding of parents who opposed vaccines in villages with high-uptake vaccination. In peripheral villages where marginalised communities have tension with the government, especially in the migrant communities, we could not discuss the more specific problems each community may have as we could invite a few migrant mothers. Another (third) limitation during data collection was our positionality towards research participants in this project. We appeared as collaborators with CDC Daklak– a governmental office, and our research team members belonged to different groups than the ethnic minority participants. Some ethnic members were reserved to talk to the research team, especially those who were not confident in speaking Vietnamese. In addition, they might be worried if they reported anything negative about governmental activities such as EPI vaccination, there could be consequences. This may have led to the overlooking of some vaccine-critical perspectives. While we tried to fill the gap by triangulating data from different sources, including village health workers in the same villages, ethnic groups in the same communes and multiple types of documents about these communities, future researchers should consider reaching these peripheral communities directly to understand their lived experiences related to childhood vaccination. It is particularly essential that future research should enable a space where the marginalised communities feel comfortable sharing their challenges and needs.

## Conclusions

In this paper, we presented a complicated picture of mothers’ views and practices about childhood vaccines in a rural multi-ethnic province in the Central Highlands of Vietnam. Using the SAGE’s matrix of vaccine hesitancy determinants, we showed that the perceptions and behaviours related to children’s vaccination are influenced by multiple social and contextual factors beyond the individual intentions. We also identified that the disparity in vaccination coverage is often observed in peripheral indigenous and migrant communities, who may have more difficulties taking children to vaccination due to financial difficulties and disconnections from the mainstream commune and healthcare workers. These patterns of vulnerability are rooted from the legacy of national wars and mass migration movement to the province after Independence, which have produced inequalities in resources and labour capacity of these peripheral communities, while inadvertently disrupting social dynamics in the province. These findings implicate the need for tailoring intervention strategies that specifically address the socioeconomic disadvantages and strengthen the social dynamics of these communities to facilitate easier vaccine decisions.

## Electronic supplementary material

Below is the link to the electronic supplementary material.

Supplementary Material 1

Supplementary Material 2

Supplementary Material 3

## Data Availability

Due to the concern of participants’ privacy, qualitative data in this manuscript cannot be made public but excerpts may be made available from the corresponding author on reasonable request.

## Abbreviations

LMIC: Low- and middle-income countries
EPI: Expanded Programme on Immunization
IDI: In-depth interview
FGD: Focus group discussion
