# Supplementary material for: High immunisation coverage but sporadic outbreaks of vaccine-preventable diseases: the structural gaps in vaccination uptake in central highlands, Vietnam
Source: BMC Public Health. 2025 Jul 3;25:2293. doi: 10.1186/s12889-025-23486-6 (PMC12225764; doi:10.1186/s12889-025-23486-6)
Supplement: Supplementary file 1 — Supplementary Material 1 [file 12889_2025_23486_MOESM1_ESM.docx]

**06EP In-depth interview**

# Demographic information (Ask all participants):

Participant Number:

Age (Year of birth):

Occupation:

Ethnic group:

How many children have you had? (Live births)

# Questions about the household

1. How long has your family/household lived in this area?

2. If your household/ or some of your household have moved in the past 10 years – Where did they move from?

# Questions about Your Pregnancies (3-28)

3. How many pregnancies have you had?

4. How many live births have you had?

5. Ask approximate ages of her children

6. Are you pregnant now?

□ yes □ no (If no, continue with Qu 10)

7. If yes, how many months into the pregnancy?

8. When you were last pregnant, or during this pregnancy, do you remember having a pregnancy check-up by a health worker? If she answers no or not sure/ can’t remember continue with Q.10

9. Where did you have the pregnancy check?

Use this list as prompts

Hospital / Private clinic / Clinic / Pharmacy / Traditional Med Clinic / Other

10. Do you know that you can have a pregnancy check?

□ Yes (If yes, continue with Q11) □ no (if no, continue with Q13)

11. Do you remember who told you about the pregnancy checks and how to get them? Explore how she receives health information.

12. If you know about Pregnancy Checks but were unable to get one, what are some of the reasons? *Open question, but use prompts if needed:*

□ Difficult travel (*Explore reasons - distance, no transport, and weather/road conditions, no one to drive her…)*

□ Did not know where to go

□ Forgot calendar

□ Do not want to go  *Explore reasons*

□ Other, _________

13. Did you remember having a vaccine during the most recent pregnancy? If she was vaccinated, ask the next questions 14. If she was not, or can’t remember continue with Qu. 21.

14. Do you remember what the vaccination was for?

15. Where did you have the vaccine?

Use prompts if needed: Hospital/ Clinic/ Other

16. How many times were you vaccinated in that pregnancy?

□ one □ two □ three □ not sure/ can’t remember

(prompt – Did you get the 3rd dose after the baby was born?)

17. Were you vaccinated in any previous pregnancy?

□ yes □ no □ not sure/ can’t remember

18. Interviewer please collect details of previous vaccinations in other pregnancies (approximate dates, how many times in each pregnancy, do they know what the vaccine was for?)

………………………………………………………………………………………………………………….

19. When you had a vaccination did you notice any reaction or effect afterwards?

□ yes □ no □ not sure/ can’t remember

20. If yes, what was the reaction? What did you do about it? What do you think about it now?

**About the most recent successful pregnancy only**

1. Where did you give birth to your child?
2. Who helped you when you gave birth?
3. Did you have your child vaccinated?

□ yes □ no ( if No, continue Q.28) □ not sure/ can’t remember

1. If yes, where did you go to have your child vaccinated?

□ Hospital □ Other, _______________

□ Clinic

1. If yes, how many times was your child vaccinated?

□ one □ two □ three □ not sure/ can’t remember

26. When your child had a vaccination did you notice any reaction or effect afterwards?

□ yes □ no □ not sure/ can’t remember

27. If yes, what was the reaction? What did you do about it? What do you think about it now?

1. Open question – explore why they did or did not have the child vaccinated.

…………………………………………………………………………….

**D. Health seeking behavior (Qu 29-34) (Ask all participants)**

1. Where did you first find out about vaccines (For you or your child)?

*Open question, but use prompts if needed:*

□ I don’t know about vaccinations

□ Health worker informs at home

□ Notified at the clinic

□ Listen to TV, radio, newspapers

□ Other, ________________________

1. If you know about vaccinations but were unable to get vaccinations for you or your child, what are some of the reasons?

*Open question, but use prompts if needed:*

□ Difficult travel (*Explore reasons - distance, no transport, weather/road conditions, no one to drive her…)*

□ Did not know where to go

□ Forgot calendar

□ Do not want to go  *Explore reasons……………………………*

□ Other, _________

1. When your child is sick, what do you do?

*Open question, but use prompts if needed:*

□ Hospital □ Private clinic visit

□ Government clinic □ the pharmacy

□ Traditional Med Clinic □ Elders

□ To the healer □ Use herbal medicine

□ Nothing

□ Other, ________________________

1. What is the MAIN reason you would choose to visit this provider for your child instead of a different place? *Tick 1 or 2*

*Open question, but use prompts if needed:*

□Close to home

□Low cost

□Trust in providers/high quality of care

□Availability of drugs

□Availability of female provider

□Recommendation or referral

□Friendly staff

□Other (specify)

□Don't know

1. When **you** are feeling sick, what do you do?

*Open question, but use prompts if needed:*

□ Hospital visit □ Private clinic visit

□ To the government clinic □ To the pharmacy

□ Traditional Medicine Clinic □ Ask my elders

□ To the healer □ Use herbal medicine

□ Nothing

□ Other, ________________________

1. What is the MAIN reason you would choose to visit this provider when YOU are sick instead of a different place?

*Open question, but use prompts if needed:*

□Close to home

□Low cost

□Trust in providers/high quality of care

□Availability of drugs

□Availability of female provider

□Recommendation or referral

□Friendly staff

□Other (specify)

□Don't know

**E. Some attitudinal questions (Qu 35-37): (Ask all participants)**

1. In your opinion, what is a vaccination? (Open question – use prompts if necessary)

□ To prevent diseases/ sickness in the future (protection

□ To cure diseases/ sickness that a person has (treatment)

□ To make you stronger (but don’t know how)

□ Don’t know

□ Never heard of it

*Please tell us if you agree with these statements:*

1. In this community, the majority of people understand what vaccines are for. (Open question – use prompts if necessary)

□Strongly disagree

□Disagree

□Neither agree nor disagree

□Agree

□Strongly agree

□Don’t know

1. In this community, the majority of people think that vaccines are a good thing.

(Open question – use prompts if necessary)

□Strongly disagree

□Disagree

□Neither agree nor disagree

□Agree

□Strongly agree

□Don’t know
