# Supplementary material for: High immunisation coverage but sporadic outbreaks of vaccine-preventable diseases: the structural gaps in vaccination uptake in central highlands, Vietnam
Source: BMC Public Health. 2025 Jul 3;25:2293. doi: 10.1186/s12889-025-23486-6 (PMC12225764; doi:10.1186/s12889-025-23486-6)
Supplement: Supplementary file 2 — Supplementary Material 2 [file 12889_2025_23486_MOESM2_ESM.docx]

**06EP Focus Group Discussion**

**Introductions**

***Ask names and ethnicity of participants.***

Ask everyone to say their name and how many children they have. If they come from different hamlets – ask them to mention where their house is – not necessary if all from 1 location.

1. Ask the group to suggest all the different facilities that they could get healthcare, including ones they know about but perhaps don’t use.

2. Ask the group which facilities do most women go to with their children, and why they choose these.

3. Ask the group, if they themselves or an adult is sick, where would they go to get health care. Is their choice different for adults compared to children? Why?

4. Ask the group what they think vaccinations are for. Do they think they are important?

What is the attitude in the wider community?

**Allow a discussion and then explain that a vaccination is to PREVENT diseases, not to cure an illness, so are given to healthy people.**

5. Do they know that there are vaccines available for pregnant women, which protect the woman and her baby? Do women in their village get vaccines when pregnant?

**The next part of the discussion is about how you find out about health care.**

6. If they do know about pregnancy vaccinations, how did they hear about them?

7. What other ways of communication about health do they know about?

Are there any problems with those methods? (language, trust, literacy, access to the communication..)

**Explain that we would like to help improve the ways that health messages are given to women in these communities, and so would really appreciate their ideas about how we should do it better.**

8. Which do they think are the best ways of sharing information with women in their community? Who should do it?

**Thank the group for their time and contribution. Invite questions from them. Invite them to stay for refreshments and offer small gift for their time.**
