# Supplementary material for: High immunisation coverage but sporadic outbreaks of vaccine-preventable diseases: the structural gaps in vaccination uptake in central highlands, Vietnam
Source: BMC Public Health. 2025 Jul 3;25:2293. doi: 10.1186/s12889-025-23486-6 (PMC12225764; doi:10.1186/s12889-025-23486-6)
Supplement: Supplementary file 3 — Supplementary Material 3 [file 12889_2025_23486_MOESM3_ESM.docx]

**Semi-structured interview – community**

In-person interviews will last between 1-2 hours and may be conducted over a series of shorter interactions with the same participant, either face-to-face or online. Questions might be re-phrased, when necessary, and additional topics and probes will be included, based on the responses of the interviewees, as well as information gathered during other data collection.

In general, all questions in this guide should be asked, however, the order can be edited to make the interview more conversational.

| **Ice breaker** |
| --- |
| **Informed consent**  If first interview, conduct full consent process; if subsequent interview, remind participant about nature of study and consent process. In all cases, ask participant if they have any questions about the study or their participation in this study. |
| **Participants’ information** |
| Participant ID  Age  Occupation  Gender  Number of children |
| **Interview part A: Questions about vaccine and vaccination** |
| First we would like to explore your experience and your thoughts about vaccination in general. |
| **Part 1: Questions about knowledge and behaviors about vaccine and vaccine preventable diseases** |
| 1. Questions about vaccine behaviors: 2. Did you vaccinate yourself during pregnancy? How many times? 3. Did you vaccinate all of your children?  - If no, ask why?  1. Do you keep your children’s vaccination cards? Can you show it to me?  - If no, ask participants if her children completed the vaccination schedule? How does she know?  1. Questions about vaccination experience: 2. Can you describe to me what it is like on one vaccination time of you/your child? 3. Are there any side effects following your/your child’s vaccination? How did you handle it? 4. Are there any noteworthy things related to vaccination that you want to share with us? 5. Questions about knowledge and perception towards vaccine and vaccination: 6. Do you know what vaccine is for?   If participant answered that vaccine is to prevent diseases, ask to list examples of vaccine – preventable diseases?   1. Do you know any [insert: “other” if the participant has described some in Q2] side effects of vaccination? Do you know how to deal with these side effects? 2. Do you think vaccine is safe? Why or why not? 3. Do you have any concerns towards vaccine/ vaccination you would like to share with us? 4. Questions about perceptions towards vaccine-preventable diseases: 5. Have you ever heard of any incidence of vaccine preventable diseases (List some)? 6. Do you think you/your children are at risk of catching those diseases? |
| **Part 2: Questions about vaccine-specific factors influencing vaccination behaviors** |
| 1. Do you have any difficulties going for vaccination?   [Prompt if needed: logistics, transportation, work, children’s fever]   1. Are there any thing that makes it easy for you to go for your/your child’s vaccination? 2. Who is the one making decision about you/ your child’s vaccination? 3. In your community, do people go for vaccination for themselves/their children? 4. What do people think about vaccination? 5. Are there any people not going for vaccination? Why? Does the rest of your community say or do anything about it? 6. Do you know that vaccination is free?   [Explain that this is the government’s policy] What do you think about that policy?   1. Are there any changes in vaccination experience now compared with the past?   If yes, do you know what causes such change? |
| **Part 3: Questions about communication and information related to vaccine and vaccination:** |
| 1. What is your communication about vaccination with: 2. Your family members 3. Neighboring women 4. Colleagues at work? 5. Do your family members/neighboring women/colleagues support your decision to vaccinate yourselves/ your child?  - If yes, ask for details what kind of support  1. Can you tell me about your experience and interaction related to vaccination with: 2. Healthcare workers from health station 3. Health staff from hospitals and clinics 4. Community health workers 5. Other administrative staff in your community (e.g. Women’s Union, Population Planning, Hamlet leader, Religion leader) 6. Media: Have you read about vaccine in newspapers/ TV/on the Internet? 7. Can you rank who has the biggest influence on your vaccination decision?   (Interviewer can use cards that portray: family member, neighboring women, colleagues, healthcare workers, community workers, religious leaders, hamlet staff, etc.)   1. At the moment, where did you get the most vaccination information from? (ranking exercise - prepare cards portraying: family member, neighboring women, colleagues, healthcare workers, community workers, religious leaders, hamlet staff, TV, newspapers, the Internet, etc. and let them rank)   Would you like to receive vaccination information from a different source? Which one?  [Prompt for Q1 & Q3 if needed: the content of information, methods of communication, how do they feel about the interaction/information] |
| **Part 4: Questions about wider factors about social dynamics and policy levels** |
| 1. How often do you communicate with your neighbors? [Prompt if needed: the content of information, methods of communication, how do they feel about the interaction or information] 2. How often do you attend social gatherings?  - If yes, which content/ occasion? - If no, why?  1. Do people in your area often talk to each other?  - If yes, what topics? - If no, why?   [Ask specifically about communication among neighboring women if not mentioned)   1. On what occasions do people in your area often meet and socialize with each other?   [Prompt if needed: social hangout, health activity, community meetings, festivals, weddings/funerals, religious events]   1. If somebody in your community wants to announce something, how would they do it? 2. Is anyone excluded from such social gathering? Do you know why? 3. Do you and your community interact with people from other ethnicities living in the same hamlet/commune? How?  - And if not, why?  1. Has there been any changes in the way your community socialize with other in recent years?  - If yes, how? And do you know what causes these changes?  1. Questions about health communication in general: 2. At the moment, where did you get the most health information from? (ranking exercise - prepare cards as Q1 and let them rank)    1. If there is a different way that you think it’s better, would you choose a different card? 3. If you use the Internet, how would you search for the information? How do you decide the websites that you look at? Do you trust the information on the Internet? 4. If the health workers at the station want to contact you or the community, how would they do it? Do you think it’s a good way to communicate with you or the community? Is there a better way? 5. Questions about health/social policies, particularly for ethnic minorities:   a. The government offers free health measures in some cases. Are you receiving any free health measures?  b. Are you receiving any other social benefits from the local authority? (E.g. financial aids, school tuition exemption, training support for job skills, etc.)   - Do you think those policies support your life? Your health seeking behavior? |
| **If this interview ends here, interviewer please refer to wrap up section and inform the participant that there will be a second interview for PART B.** |

| **Interview part B: Questions about experience of COVID-19 experiences** |
| --- |
| Now [Insert: “Today” if this is a follow up interview to the above], I would like to know about your experience about COVID-19  First, can you tell me Have you been tested for COVID-19?  If yes, what was the results? |
| **Part 1:** **Open ended narrative of COVID-19 experiences** |
| [*Goal: to gain a narrative of experiences from the participant’s point of view prior to any focused questions to gain a picture of what their experiences include.]*   1. To start, we would like to know more about your experiences during the COVID-19 outbreak. After, we will ask more detailed questions but to begin, please tell us the story of your life during COVID-19. Please start the story where you like and take as much time as you need. 2. [*when they are finished*] Thank you for sharing your story. I would like to ask you more about [*insert 1-2 questions that you would like to probe on from their story*].   [*probe: during the narrative, try not to interrupt for details – note any questions you would like to probe on, use acknowledgement probes, e.g. ok, yes, mhhh and gentle probes, e.g. “is there anything else you would like to add” or “what else happened?” and then ask follow-up questions after.]* |
| **Part 2: How is COVID-19 understood in different locales?** |
| Topics: COVID-19 knowledge, health care seeking changes, information     1. Could you tell me more about what you know about COVID-19?    1. [*potential probes: where did it come from? How does it spread? Is it dangerous? Who gets it? Is anyone at more/less risk to acquire it? Add additional probes from survey results]* 2. What are the symptoms of COVID-19? 3. Before the COVID-19 outbreak, what would you do if you had similar symptoms to those you just listed? 4. Has that changed since COVID-19?    1. Why or why not?    2. *[if yes]* Could you elaborate on how it has changed? 5. Where do you and your family obtain information about COVID-19? [*probes, if necessary*: *TV, social media (Facebook, Zalo), radio, friends, family, healthcare professionals]* 6. Could you tell me more about the types of information provided from these sources? [*probe: What are some of the exact messages or information (‘facts’) that you remember from these sources?]* 7. Are there sources that are not trusted by the community in general? If yes, how do you know if the information is good or bad (or true or ‘fake’)? [*probe about any survey or media results]* |
| **Part 3: Disease transmission, adherence to public health guidelines, and ad hoc harm reduction** |
| We have a few questions about various measures you and your family have taken in response to COVID-19. By family, for these questions, we mean those who are living in your home with you.   1. Who all is living in your home right now? [*probe: # of people, generations*]    1. Has this changed since before COVID-19? If yes, how and why? 2. What are the three biggest changes that have occurred for you and your family since COVID-19 (or during COVID-19)?    1. [*probe for details on specific situations, for each challenge]* Did you have these challenges prior to COVID-19?    2. *[if had challenges before]* Has this challenge become better or worse for you and your family? Why and how? 3. What do you think you should you do to protect yourself and your family during this outbreak or future outbreaks? [*probe: for each method, ask their perception of the effectiveness and how easy or difficult it is for them and their family to do it and why*] 4. [if not discussed in q12] What was it (or is it) like **to isolate at home**? Was there anything that made it more difficult or easier for you and your family? If yes, what were they? [*probe: work related, care taking responsibilities outside the home, family interaction, family relationship dynamics, etc*]. 5. How did **the isolate at home** order change your practices for events that typically involve a social gathering? (*probe if necessary e.g. religious activities, weddings, funerals, births, graduation, other common rituals for that society*).    1. Could you give an example of how you changed your activities related to these events? 6. How did/does the COVID-19 and the public health response impact your daily life? [*probe about livelihoods, healthcare seeking (routine + vaccinations*)] 7. [*if active or past contact tracing in community*] What do you think about the contract tracing that governments are conducting in order to identify contacts of those with confirmed COVID-19?    1. Do you think this is an effective measure? Why or why not?    2. Do you think it is an invasion of personal privacy? 8. [*if active or past contact tracing in community*] Have you or a family member been involved in contact tracing: either had to provide information on your contacts or have been informed that you were potentially exposed through a contact?    1. If yes, what was that experience like? |
| **Part 4: Social stigma and ‘othering’ associated with COVID-19** |
| 1. Are there specific groups who are more responsible for the spread of COVID-19 in [*insert country*] or more broadly?    1. [If yes] Who are these groups?    2. [If yes] Why do you think they are more responsible for the spread of COVID-19? 2. Do you think *all* health care workers and health care staff should temporarily live in a space away from their families during COVID-19? Why or why not? 3. [*if not mentioned*] Do you think health care workers who are working at quarantine facilities or hospitals with known COVID-19 cases should temporarily live in a space away from their families? Why or why not? 4. [*add country specific questions, as appropriate*] |
| **Wrap Up** |
| Is there anything else you would like to add to our discussion related to your experiences of vaccination/COVID-19?  If not, this interview ends here. Thank you very much for your time and your answers. |
